# Laser driven FLASH radiobiology using a high dose and ultra high dose rate single pulse proton source

**DOI:** 10.1038/s41598-025-01105-z

**Published:** 2025-05-13

**Authors:** A. Flacco, E. Bayart, L. Romagnani, M. Cavallone, L. De Marzi, C. Fouillade, C. Giaccaglia, S. Heinrich, I. Lamarre-Jouenne, J. Monzac, K. Parodi, A. Patriarca, T. Rösch, J. Schreiber, L. Tischendorf

**Affiliations:** 1 Laboratoire d’Optique Appliquée, ENSTA Paris, CNRS, Ecole Polytechnique, Institut Polytechnique de Paris, 91120 Palaiseau, France; 2 LULI, CNRS, École Polytechnique, Institut Polytechnique de Paris, 91120 Palaiseau, France; 3 Laboratoire d’Optique et Biosciences, Ecole Polytechnique, CNRS, INSERM, Institut Polytechnique de Paris, Palaiseau, France; 4 Department of Radiation Oncology, Campus Universitaire, Institut Curie, Centre de Protonthérapie d’Orsay, PSL Research University, 91898 Orsay, France; 5 INSERM LITO 1288, Campus Universitaire, Institut Curie, PSL Research University, University Paris-Saclay, 91898 Orsay, France; 6 Institut Curie, INSERM U1021-CNRS UMR 3347, Paris Saclay University, Centre Universitaire, 91405 Orsay Cedex, France; 7 Institut Curie, PSL Research University, 75006 Paris, France; 8 Department of Medical Physics, Faculty of Physics, Ludwig-Maximilians-Universität München, 85748 Garching bei München, Germany

**Keywords:** Laser-driven proton acceleration, Ultra-high dose-rate, FLASH, Quadrupoles, Dosimetry, Laser-produced plasmas, Preclinical research

## Abstract

Laser-driven proton sources have long been developed with an eye on their potential for medical application to radiation therapy. These sources are compact, versatile, and show peculiar characteristics such as extreme instantaneous dose rates, short duration and broad energy spectrum. Typical temporal modality of laser-driven irradiation, the so-called *fast-fractionation*, results from the composition of multiple, temporally separated, ultra-short dose fractions. In this paper we present the use of a high-energy laser system for delivering the target dose in a single nanosecond pulse, for ultra-fast irradiation of biological samples. A transport line composed by two permanent-magnet quadrupoles and a scattering system is used to improve the dose profile and to control the delivered dose-per-pulse. A single-shot dosimetry protocol for the broad-spectrum proton source using Monte Carlo simulations was developed. Doses as high as 20 Gy could be delivered in a single shot, lasting less than 10 ns over a 1 cm diameter biological sample, at a dose-rate exceeding $10^{9}\hbox { Gy s}^{-1}$. Exploratory application of extreme laser-driven irradiation conditions, falling within the FLASH irradiation protocol, are presented for irradiation in vitro and in vivo. A reduction of radiation-induced oxidative stress in vitro and radiation-induced developmental damage compatible with the onset of FLASH effect were observed in vivo, whereas anti-tumoral efficacy was confirmed by cell survival assay.

## Introduction

Radiation therapy is a cornerstone in cancer management. Apart from X-rays, which represent the strong majority of treatments, other radiation qualities and disparate spatial or temporal source parameters are used to match particular therapeutic needs. Unlike X-rays, protons have a rapid distal dose fall-off and a reduced proximal dose.

Laser-driven proton sources have been proposed as a promising alternative to conventional (cyclotrons, synchro-cyclotrons) accelerators, either for deep^1,2^ or superficial^3^ treatments. Lasers, as an energy source, do not require specific radioprotection before interaction with matter; moreover ion plasma sources produce broad spectra, which could offer novel alternative strategies for producing a spread-out Bragg peak (SOBP), used for the treatment of deep-seated tumors. As of today, existing laser technologies and explored acceleration strategies do not provide sufficient kinetic energies for the medical application, although successful in vitro^4,5^ and surface in vivo^6^ irradiation experiments made a huge step forward to demonstrate their practical use in radiation biology.

The recent discovery of the FLASH effect by Favaudon et al.^7^ renewed the interest around the role of dose-rate and temporal modality of dose deposition in defining the biological and the physiological effect of ionizing radiation. Some advantages demonstrated for in vivo and human irradiation (see Wilson et al.^8^ and references therein for a consistent review) suggest a possible improvement of the therapeutic window at high dose rate, which motivates the exploration of non-conventional temporal dose deposition modalities. Laser-driven particle sources produce short and bright particle bunches, as each laser pulse independently extracts and accelerates a short and bright packet. Owing to the ultra-fast nature of laser pulses at relativistic intensity, laser-accelerated protons have a duration, at the source, between few picoseconds and few nanoseconds^9,10^.

An ever increasing number of laboratories worldwide^11–14^ are exploring the biological effects of laser-accelerated protons, where relevant irradiation conditions are met in terms of particle penetration, irradiated volume and average dose-rate. Such systems are often limited to an energy-per-pulse of a few to tens of joules and repetition rates in the Hz range or lower. The total useful proton charge from such systems is sufficient to deposit a dose of a few mGy to a fraction of Gy per shot, where a projected target surface of ${1} {\hbox { cm}^{2}}$ is considered. In this condition, multiple laser pulses are required to produce a target dose within 1 to 10 Gy, with a total irradiation time not shorter than a few seconds and up to a few minutes. This irradiation modality, where the dose is deposited by separate fractions at ultra-high dose-rate, termed “fast fractionation”, was shown to produce particular biological effects in some conditions^4,15^. In a recent experiment^16^, the use of laser-driven electron source for very-high dose, single pulse irradiation was also demonstrated.

In this paper we present the use of a kJ-class laser to achieve the total target dose within a single laser-driven proton pulse. A set of two permanent-magnet quadrupoles is used to transport and shape the protons to an irradiation area in air, far from the interaction point. This technique gives access to doses as high as 20 Gy delivered within a few nanoseconds over a ${1} \hbox { cm}^{2}$ *in vitro* sample.

The transport line provides an additional control over the irradiation conditions, such as the ability to vary the projected charge density in the proton bunch, for performing dose-escalation experiments. Moreover, the spectral selection operated by the quadrupoles can be used to produce a uniform depth dose deposition (SOBP) within thicker targets (up to ${600}\,\upmu \hbox {m}$ of water).

In order to correctly apply our particle source to radiobiology experiments, a dosimetry protocol for the reconstruction of the spectral content of each laser shot is developed and applied to all of the explored irradiation conditions.

A survival assay of human glioblastoma cell line U87-MG is performed as a confirmation of the toxicity to cancerous cells of the irradiation protocol. The generation of stress-dependent oxydative stress is studied in vitro in healthy (MRC5) and tumoral (U87-MG) cell lines, showing reduced DNA damage in the healthy cells. This suggests a protective effect of the high-dose-rate irradiation condition. Evaluation of zebrafish embryos development following irradiation under these conditions suggest onset of the FLASH effect with short, laser-driven, single pulse proton irradiation scheme.

## Source and transport

**Fig. 1:**
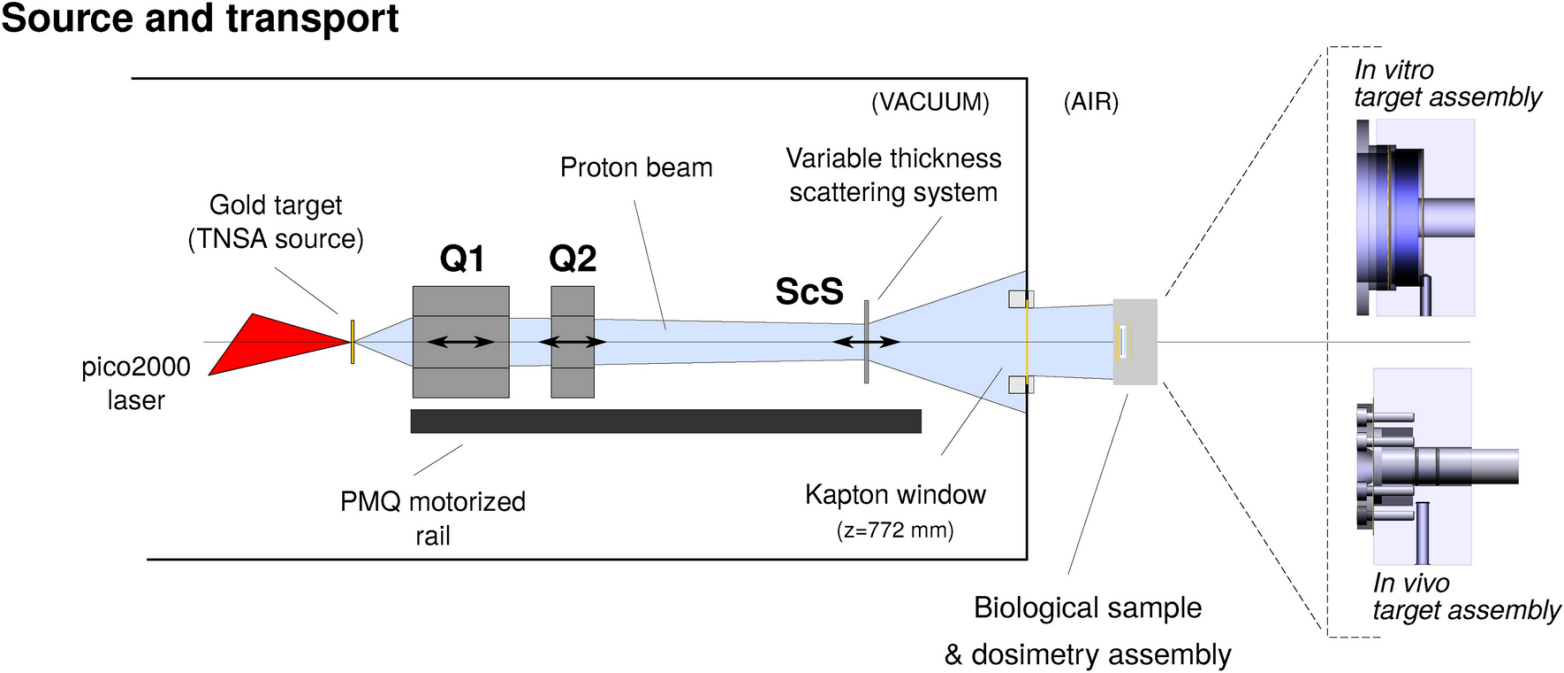
Scheme of the experimental setup, showing the TNSA proton source, the transport line composed by two permanent-magnet quadrupoles (Q1, Q2), and the scattering system (ScS), all contained within a vacuum, along with dedicated assemblies for the irradiation of biological samples.

The experiment was performed in two separate campaigns at the LULI laboratory (École Polytechnique, Palaiseau, France) on the *pico2000* laser system. The picosecond beam (duration ${1.2}{\hbox { ps}}$, energy 100 J per pulse, $\lambda = {1053}\hbox { nm}$) is focused by a *f*/4.4 off-axis parabola (focal length $f={800} \hbox { mm}$, diameter $d={180}\hbox { mm}$).

Protons are accelerated in the target-normal sheath acceleration (TNSA) scheme. Thin gold foils are glued on a rigid metallic holder which enables up to twenty-one independent targets to be installed and shot before venting. Optimal acceleration conditions, showing both highest charge and cut-off energy, were determined from previous experimental campaigns (Fig. 11). The TNSA mechanism relies on the energy transfer from the laser-heated electron fraction to the cold ion plasma, during the plasma expansion in vacuum (see Macchi, 2017^17^ and references therein); ion spectrum is exponential with a high-energy cutoff. In order to characterize the particle source for magnetic transport design, a set of stacked radiochromic films (RCF) of the type HD810 is used. The stack is installed at $z={48} \textrm{mm}$ from the interaction point ($z=0$) and irradiated with protons from a single laser shot. The spectrum and total charge in the accelerated beam were extracted from irradiated RCF stack following the protocol proposed by Breschi et al.^18^.

**Fig. 2:**
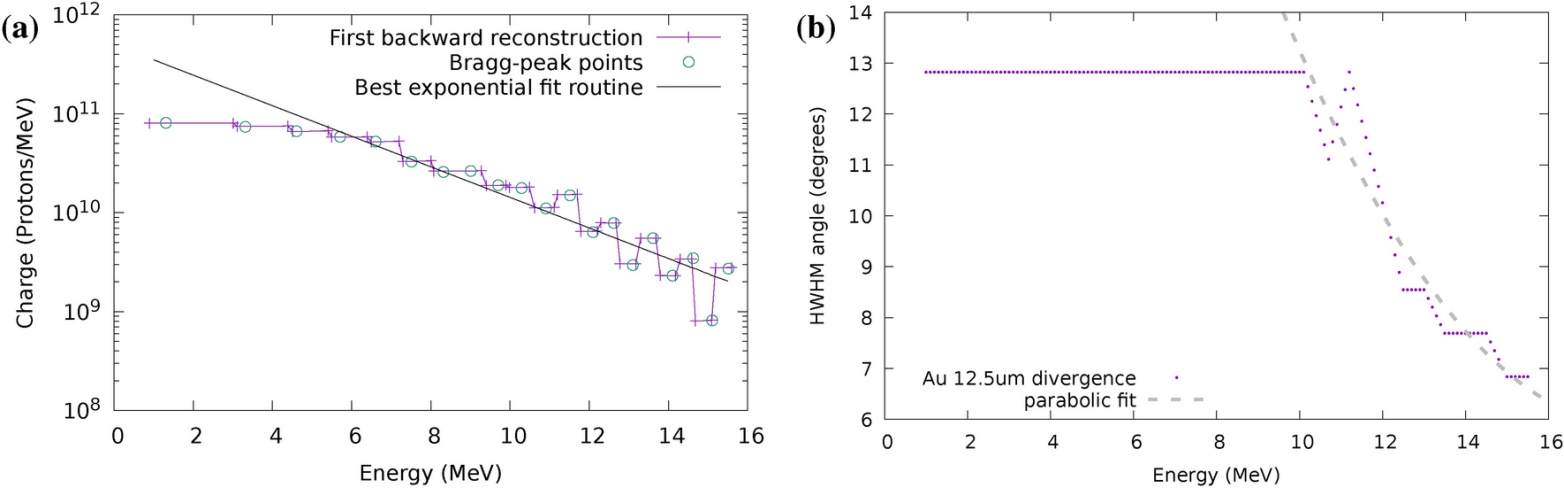
Spectral charge **(a)** and spectral divergence **(b)** on a single calibration shot, reconstructed from a stack of 25 HD810 radiochromic films. Circular dots on panel (**a**) indicate the Bragg peak energy within each foil on the stack. Foils #1 through #4 are saturated; foils #20 through #25 were not exploitable for the fitting procedure and only used to assess the cut-off energy.

A calibration shot to assess the charge at the source and its spectral features is illustrated in Figure 2a for a ${12.5}\,{\upmu \textrm{m}}$ thick gold target. The spectrum exhibits the expected exponential (thermal) profile, with a cutoff at 19.6 MeV. Radiochromic films #1 through #4 were saturated, possibly because of the contribution from heavier ions (see Methods). Different spectral components exhibit a decreasing divergence at increasing energy (Figure 2b). It’s interesting to note that protons with energy $\textrm{E}<{10}\hbox { MeV}$ are emitted with constant divergence, producing a rather sharp boundary on the radiochromic films deeper in the stack. At higher energies, $\textrm{E}>{10} \hbox { MeV}$ the beam divergence decreases following a parabolic profile. According to the general calibration function of the HD810 film, the total charge within the exponential fit for the presented shot exceeds 150 nC.

### Transport configuration

The transport line is designed to produce a controlled irradiation to a plane outside the experimental chamber. Air-vacuum separation is guaranteed by a ${75}\, \upmu \textrm{m}$ thick Kapton film, situated at $z={772}\,{\textrm{mm}}$ from the interaction point, whereas the irradiation plane lays at $z={827}\,{\textrm{mm}}$ (Figure 1). The transport line is composed by two permanent-magnet quadrupoles and a variable-thickness scattering filter. Quadrupoles Q1 and Q2 are respectively $d_1={40}\,{\textrm{mm}}$ and $d_2={20}\,{\textrm{mm}}$ long, and have field gradients of $g_1={332} \hbox { T m}^{-1}$ and $g_2={322} \hbox { T m}^{-1}$. Quadrupoles construction and calibration is detailed in Rösch et al.^19,20^. Both quadrupoles have an inner bore of 1 cm diameter; considering the shortest possible distance between quadrupole Q1 and the proton source, $\Delta z= {48}\hbox { mm}$, the bore sets a maximum angular acceptance of $\theta _{max} = {100}\,\hbox {mrad}$. The scattering system (ScS) is composed by four different selectable aluminum filters of varying thickness to be inserted between the quadrupole Q2 and the Kapton window, to provide diffusion of the proton beam, improving divergence and transverse uniformity. All elements on the transport line are motorized and equipped with optical encoders, enabling reliable positioning even in presence of strong magnetic forces between the elements.

The role of the transport line is to control the particle density and, consequently, the deposited dose at the target plane, far from the laser-plasma interaction point. During the design step the deposited dose at the irradiation plane is simulated by Monte Carlo methods (Geant4 toolkit) for different beam line configurations. The source is modeled as being purely exponential (thermal) with a high energy cutoff and no angular dependence. This assumption is based on the small effective aperture of the first quadrupole, which acts as a filter on the broad divergent source. The largest accepted half-width angle ($\theta _{max} = {6}^{\circ }$) is, according to measurements in Fig. 2, smaller than the source divergence at the cutoff energy. For this reason we decided to drop the angular dependence in the source modelling; the useful spectral charge is then represented analytically as:

1 $$\begin{aligned} Q^{*}\left( E \right) \textrm{d}E= \frac{Q_0^{*}}{E_0} \exp \left[ - E /E_0 \right] \textrm{d}E, \end{aligned}$$

where E is the proton kinetic energy, $\hbox {E}_0$ the spectral distribution temperature and $Q^{*}$ a charge. The term $\hbox {Q}_{0}^{*}$ (measured in $\text {nC}^*$ from now on to avoid confusion) indicates that the integrated charge of the source term expressed in Eq. (1) differs from the total charge at the laser-plasma source, as it is limited to the charge emitted within the first quadrupole input acceptance angle (see Monte Carlo simulation section in Methods for further details).

Two sets of configurations are designed for the particle transport. A first condition (termed *dose escalation*, DE) is studied for irradiation in vitro. The surface to be irradiated is set to have a diameter of 1 cm, centered on axis on a Lumox®capsule and the biological sample represented by a ${20}\,{\upmu \hbox {m}}$ thick water volume. In this condition the quadrupole Q2 is set at 20 mm from the first quadrupole.

**Fig. 3:**
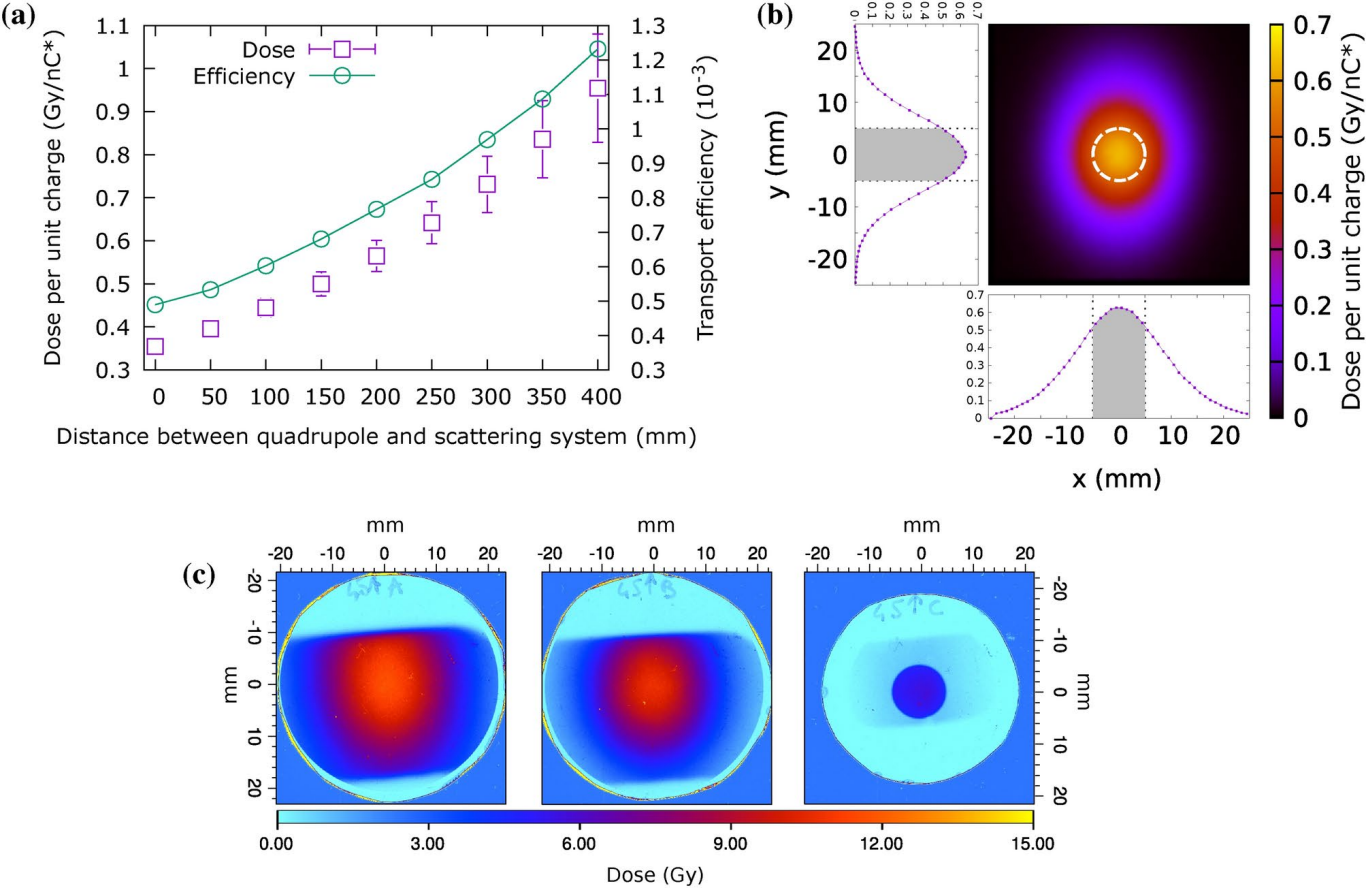
Deposited dose in the water sample and transverse uniformity at the irradiation plane for thin biological samples (${20}\,{\upmu \textrm{m}}$ water) in the dose escalation (DE) configuration. **(a)** Simulated dose and transport efficiency per unit collected charge for varying distance between Q2 and ScS, with a ${70}\,{\upmu \textrm{m}}$ aluminum scattering filter. **(b)** Simulated dose map corresponding to a dose escalation configuration with filter at $\Delta z\left[ ScS-Q2 \right] ={275}\,\hbox { mm}$. **(c)** Experimental dose map recorded with the sample holder in place in the same transport configuration as **(b)**.

In this configuration the scattering filter holds a ${70}\,{\upmu \textrm{m}}$ aluminum foil and is moved to vary the particle density at the irradiation plane, hence the deposited dose within the projected surface (Fig. 3). The transport efficiency, defined as the ratio between primary particles reaching the water sample within a 10 mm region-of-interest (ROI) and $Q_0^{*}$, varies following the distance of the scattering filter as shown in Fig. 3a.

**Fig. 4:**
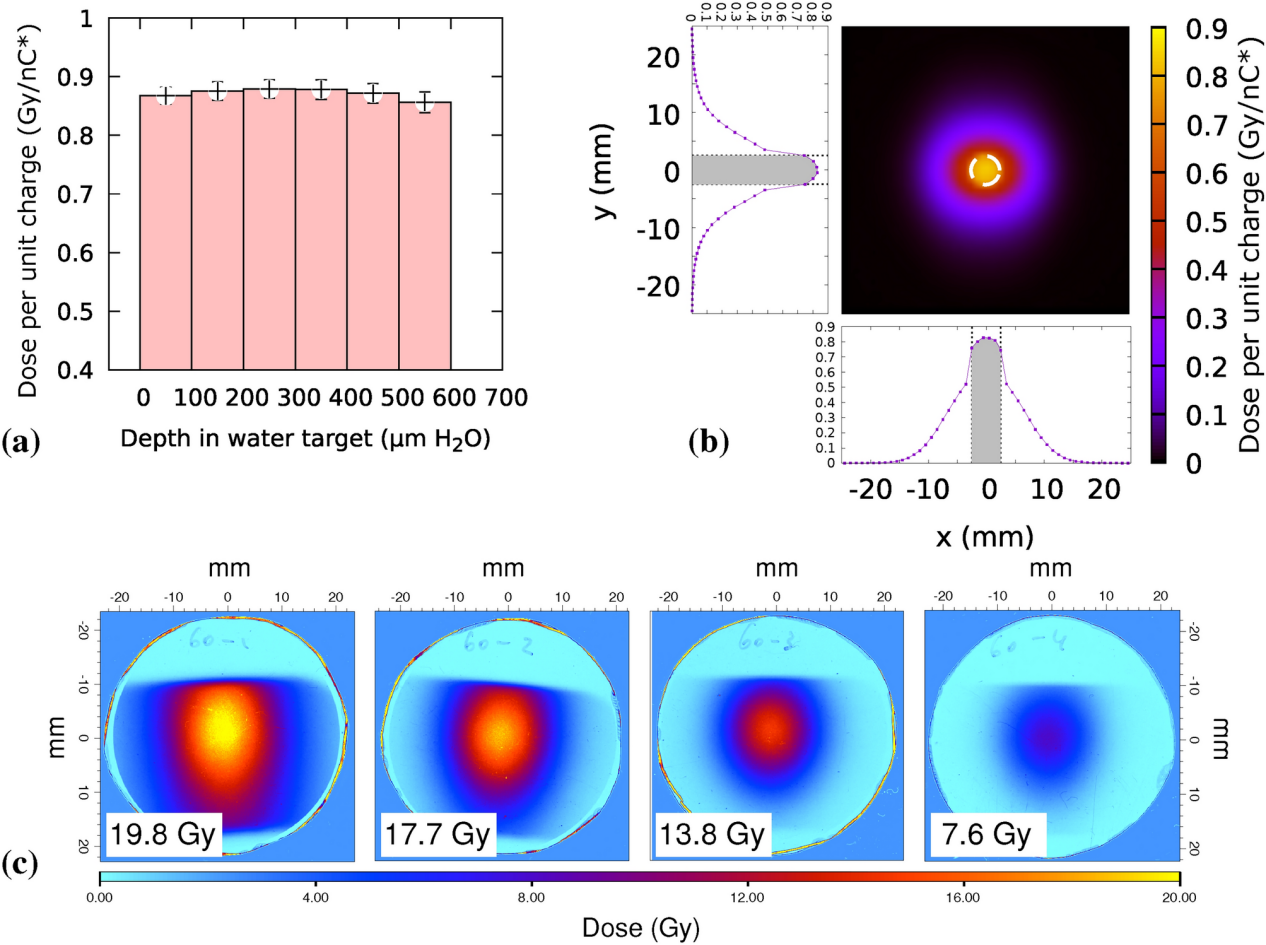
Deposited dose in a thick sample (${600}\,{\upmu \textrm{m}}$ water) in the ZF configuration. **(a)** Dose per unit input charge at varying depth in water with $\Delta z\left[ ScS-Q2 \right] ={250} \hbox { mm}$ and a scattering filter of ${50}\,{\upmu \textrm{m}}$ aluminum. **(b)** Simulated 2D map of integrated dose through the entire sample. **(c)** Experimental dose maps recorded on a test shot where EBT-XD type radiochromic films were inserted in the embryos confinement volume (see Fig. 16) verify penetration. Each RCF has water equivalent thickness of ${385}\,{\upmu \textrm{m}}$. Inset doses refer to the 5 mm diameter ROI marked in panel **(b)**.

An additional configuration was studied for irradiation *in vivo* (Zebrafish embryos, ZF). In this condition the target dose at the sample is set to be as close as possible to a well defined condition (8 Gy, as in Bourhis et al.^21^) while keeping the maximum in-depth (SOBP) uniformity. This irradiation condition was obtained with an aluminum scattering filter of ${50}\,{\upmu \textrm{m}}$ positioned at a distance of 250 mm past the quadrupole Q2; in this configuration a narrower beam is produced, and consequently a higher dose deposited within the ROI at the irradiation plane, as depicted in Figure 4. The ROI is here limited to a diameter of 5 mm.

## Single pulse irradiation and dosimetry

In our experimental condition, the target dose is deposited by the charge driven by a single laser pulse, within a time shorter than 10 ns. During irradiation it is not possible to monitor or regulate the deposited dose with a monitor chamber, as performed during previous experiments^5^. Spatially resolved dose maps are recorded by calibrated EBT-XD radiochromic films as a reference to the dose in the target sample. However, the width of the proton spectrum and the low overall kinetic energy in the beam make the energy loss in the RCF itself non negligible. Consequently, not only the dose at the RCF differs from the dose at the target sample, but the sole presence of the RCF does modify the target irradiation conditions. The shot-to-shot variation of the laser-driven proton source parameters (energy, charge) we observed did not enable to establish one irradiation condition within an acceptable error margin beforehand. Laser-driven proton acceleration in the TNSA scheme is in fact very sensitive to the total energy and, more importantly, to the laser-temporal contrast, which are beyond the user control. During the experimental campaigns reported here, measured average laser energy was (103.0 ± 5.5)J, whereas deposited dose in the same transport configuration could have a shot-to-shot variance as high as 30%.

In such condition, where irradiation is driven by a single laser pulse, the high variability hinders the possible definition of an average spectrum for a calibrated response of radiochromic films on the irradiation line, and imposes a self-consistent single-shot dosimetry protocol.

### TNSA proton source reconstruction

**Fig. 5:**
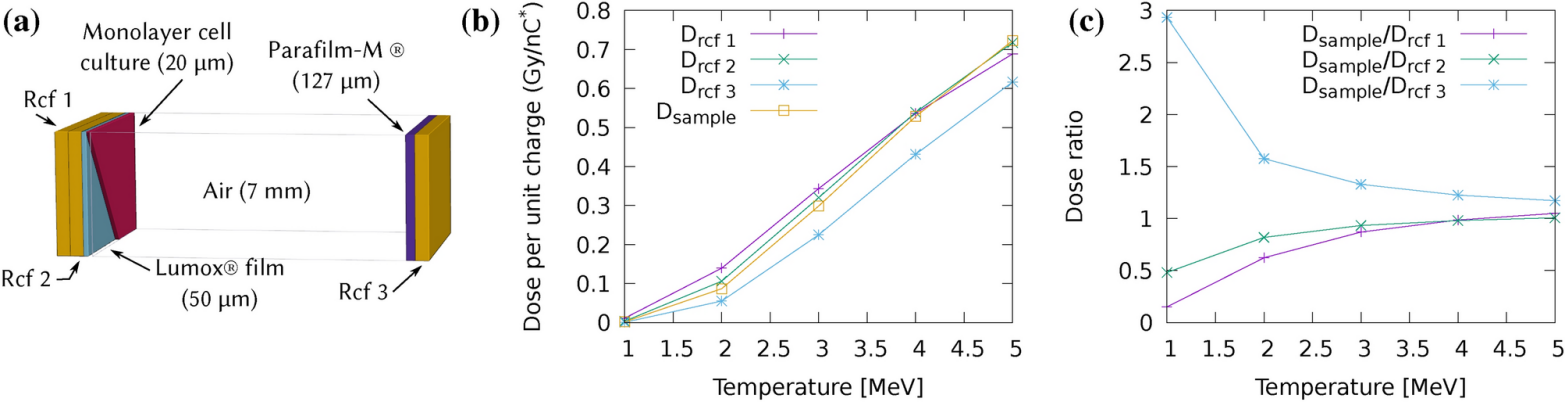
Effect of the spectral temperature on dosimeters and biological sample in the in vitro irradiation setup on the condition depicted in Fig. 3 ($\Delta z\left[ ScS-Q2 \right] ={275}\,\textrm{mm}$). (**a**) Material stack used in Monte Carlo simulation of the in vitro holder, showing the RCF position with respect to cell holder and biological sample. (**b**) Simulated dose per unit charge as a function of the spectral temperature. (**c**) Simulated dose ratio between biological sample and each of the superposed RCF in the irradiation setup.

Spectrum and charge changes at the source result in variations of the total deposited dose, as well as in the dose distribution among different planes in the irradiated volume (see for example the in vitro holder structure Fig. 5a). One fundamental assumption on the shot-to-shot behaviour of the proton source is the conservation of its exponential nature, which is well justified for the TNSA acceleration mechanism. Under this hypothesis, shot-to-shot parameter change can be considered to affect the only two free parameters in the spectral distribution (1), namely the total charge $\hbox {Q}_{0}^{*}$ and the spectral temperature $\hbox {E}_0$. A third parameter known to depend on laser conditions is the cutoff energy, $\hbox {E}_{high}$, which is usually defined as a reference parameter for laser-driven proton acceleration conditions. For our purposes, a simple rejection criterion can be set for those shots whose cutoff energy is not sufficient to penetrate the irradiated target stack. Minor variations can be neglected because, according to Monte Carlo modelling of the irradiation line, the highest energy portion of the spectrum contributes to a lesser extent to the total deposited dose, with respect to the central portion of the spectrum (see Fig. 14b in Methods). For these reasons we consider the cutoff energy constant throughout our analysis.

At least two (although often three) RCF were used in most experimental events, inserted before and after the biological sample during irradiation (see panel (a) in Fig. 5). Deposited dose in all of the RCFs and the irradiated target is simulated for the corresponding beam-line configuration and sources with a varying temperature $\hbox {E}_0$ of the exponential spectrum between 1 and 5 MeV. Following previous considerations and according to Eq. (1), total charge $\hbox {Q}_0^*$ and spectral temperature $\hbox {E}_0$ can be extrapolated from the Monte Carlo simulation which would correctly predict the dose ratio between the superposed RCF.

**Fig. 6:**
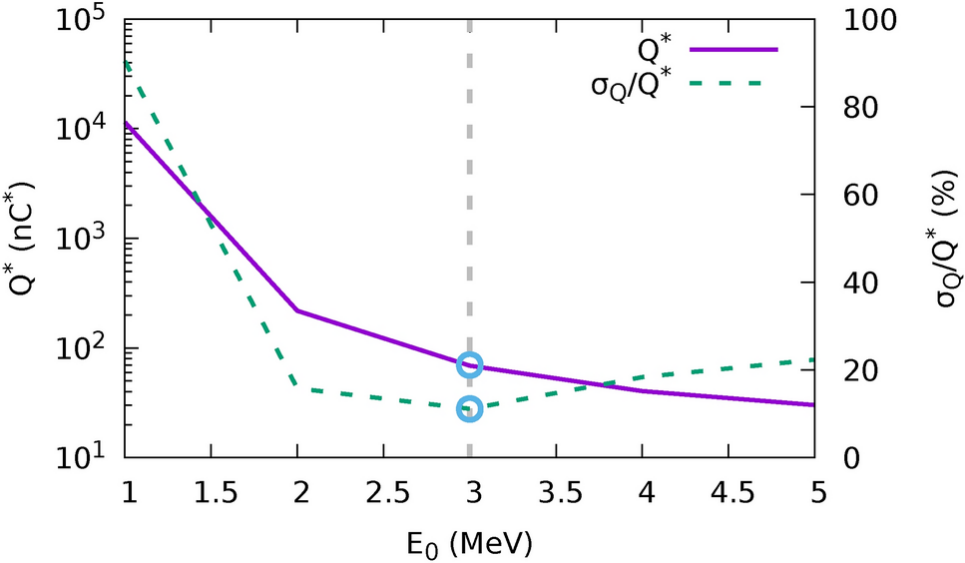
Example of charge extrapolation for one experimental event (one laser shot). Average charge over one set of radiochromic films is plotted at different source temperatures; the associated error, is used to determine the best fitting temperature. $\sigma _{E_{0}}$ is minimized at $\textrm{E}_{0}={3} \hbox { MeV}$ with an error of 11.1 %, which points to a charge of ${69.9}{\hbox { nC}^{*}}$.

Simulated deposited dose is scaled to the measured dose on radiochromic films for all the temperatures in the set. The corresponding charge, averaged for the available RCFs in a single shot, can be then expressed as:

2 $$\begin{aligned} \overline{Q_{E_{0}}^{*}}= & \frac{1}{N} \sum _{i}^{N} Q_{i,E_{0}}^{*} = \frac{1}{N}\sum _{i}^{N} \frac{D_{i}^{(rcf)}}{ d_{i,E_{0}}^{(sim)} }\end{aligned}$$

3 $$\begin{aligned} \sigma _{E_{0}}= & \frac{1}{N} \sqrt{ \sum _{i}^{N} \left( Q_{i,E_{0}}^{*} - \overline{Q_{E_{0}}^{*}} \right) ^{2}, } \end{aligned}$$

where $D_{i}^{(rcf)}$ is the experimental integrated dose on the i-th film in the target and $d_{i,k}^{(sim)} = \frac{D_{i,k}^{(sim)} }{Q^{*(sim)}}$ is the simulated dose per unit charge at the spectral temperature $\hbox {E}_0$ deposited on the i-th film. Figure 5b,c show the relationship between doses at different planes for the case of an experimental shot in the dose escalation configuration with filter at $\Delta z_{\left[ ScS-Q2 \right] }={275}\,\textrm{mm}$. From the analysis of the calculated dose ratios in the different simulations, it is possible to set the matching temperature by minimizing the standard deviation of the extrapolated charge; as an example Figure 6 depicts the minimization of the ratio $\sigma _{Q}/Q^*$ in the case of a test shot, which points to a temperature of $\textrm{E}_0 = {3} \hbox { MeV}$. The corresponding value of $\overline{\textrm{Q}_{{3\hbox { MeV}}}^*} = {69.9}\,{\hbox {nC}^*}$ is then used to rescale the simulation and assess the dose in the biological sample. The obtained values are shown in Table 1.

**Table 1:** Example of dose reconstruction of a reference shot, showing measured dose within the ROI on the three radiochromic films and the dose at the sample extrapolated through Monte Carlo simulation.

Position in the stack	RCF 1	RCF 2	**Sample**	RCF 3
Dose (Gy)	23.3	16.8	**19.2**	12.2
Quenching corrected Dose (Gy)	25.59	18.57		13.56

In some of the experimental conditions only one RCF foil was used in front of the biological target. This was the case for fish irradiation, where we aimed at preserving the best possible SOBP uniformity; adding a second radiochromic film would have reduced the penetration depth of the proton beam. Furthermore the actual design of the ZF holder does not allow the insertion of a back RCF film (see Fig. 16 for details).

**Fig. 7:**
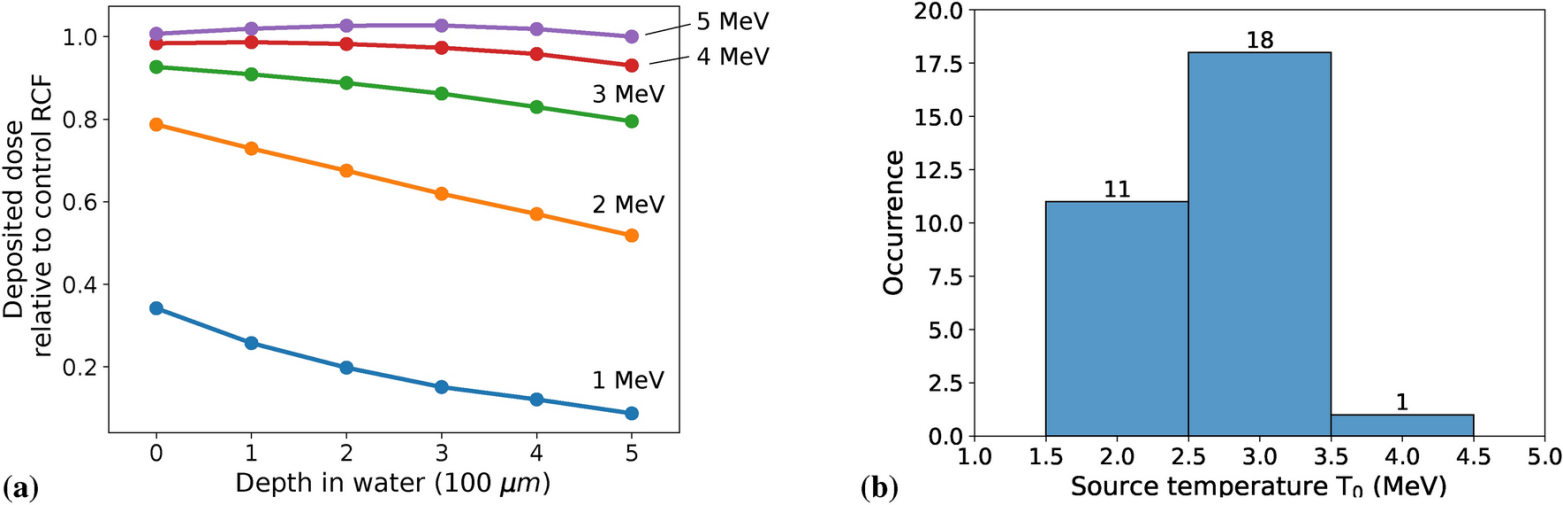
(**a**) Simulated depth-dose distribution normalized to a single RCF foil dose on top of ${600}\,{\upmu \textrm{m}}$ water target for $\hbox {E}_0$ ranging between 1 MeV and 5 MeV. Associated fit parameters are shown in Table 2. (**b**) Observed matching temperature during all of the analysed laser shot where more than one RCF was present on top of the irradiated sample.

In these cases only one experimental point is available for assessing the ratio between the deposited dose and the RCF reading, hence it is not possible to determine the parameters $\hbox {E}_0$ and $\hbox {Q}_0^{*}$ in a unique way using the previously described protocol. In order to assess the ratio between the RCF reading and the deposited dose we decided to use the temperature distribution from the entire set of in vitro events, shown in Fig. 7b as a statistical weight to the depth-dose curve and ratio to RCF depicted in Fig. 7a and detailed in Table 2.

Following this method, the quantities *sample-dose-to-RCF* and *depth-dose-error* are calculated as

4 $$\begin{aligned} \frac{D_{sample}^{exp}}{D_{rcf}^{exp}}&= \sum _{E_0} \left( \frac{n_{E_0}}{n_{tot}}\right) \left( \frac{D_{avg}}{D_{rcf}} \right) _{E_0} \left( \frac{QCF}{DWE} \right) _{E_0}= 0.77\end{aligned}$$

5 $$\begin{aligned} \frac{\sigma _{sample}^{exp}}{D_{sample}^{exp}}&= \sum _{E_0} \left( \frac{n_{E_0}}{n_{tot}}\right) \left( \sigma _{depth} \right) _{E_0} = 0.17 \end{aligned}$$

where the weights $n_{E_{0}}/n_{tot}$ (spectrum temperature occurrence probability) are represented in Fig. 7.

**Table 2:** Parameters for depth-dose distribution at varying temperature shown in Fig. 7.

Temperature $\hbox {E}_0$ (MeV)	Dose $D_{avg}$ $\hbox {(Gy/nC}^*)$	Depth-dose error (relative)	RCF Dose $D_{rcf}^{sim}$ $\hbox {(Gy/nC}^*)$	Dose ratio $D_{avg}^{sim} / D_{rcf}^{sim}$	DWE	QCF
1.0	2.36 $\times \, 10^{-3}$	1.36 $\times \, 10^{-3}$ (57.5%)	1.54 $\times \, 10^{-2}$	0.16	0.963	1.21
2.0	8 $\times \,10^{-2}$	1.94 $\times \, 10^{-2}$ (24.1%)	0.15	0.55	0.970	1.19
3.0	0.251	3.3 $\times \,10^{-2}$ (13.2%)	0.33	0.77	0.974	1.18
4.0	0.424	3.6 $\times \, 10^{-2}$ (8.5%)	0.49	0.87	0.976	1.17
5.0	0.561	3.3 $\times \, 10^{-2}$ (5.99%)	0.6	0.94	0.977	1.169

## Application to radiation biology

The irradiation beam-line was used for irradiation on several in vitro and in vivo biological targets, in order to verify its functional hypothesis, provide a rough validation of the dosimetry protocol and start to explore this novel irradiation condition.

In the following, radiation qualities are indicated as SP-LAP for “single-pulse laser-accelerated protons”, FF-LAP for “fast-fractionated laser-accelerated protons” (indicating a dose deposited by multiple separated fraction at ultra-high instantaneous dose-rate) and CAP for “conventional-accelerated protons”.

### Cell survival assay after exposure to laser-accelerated protons (LAP) at pico2000

The impact of SP-LAP is evaluated on the highly resistant glioblastoma cells of the line U87-MG, through a cell-survival assay. Cells are prepared as in a previous experimental campaign^4^ where the effect of laser-pulse pacing in a fast-fractionation irradiation modality was explored. It’s worth noting that U87-MG showed no sensitivity to the average dose-rate at fixed dose within the range explored during previous experiments (dose-per-fraction: 0.7Gy, delay between fractions: ${2}{\textrm{s}} \le \Delta t \le {60}{\textrm{s}}$).

**Fig. 8:**
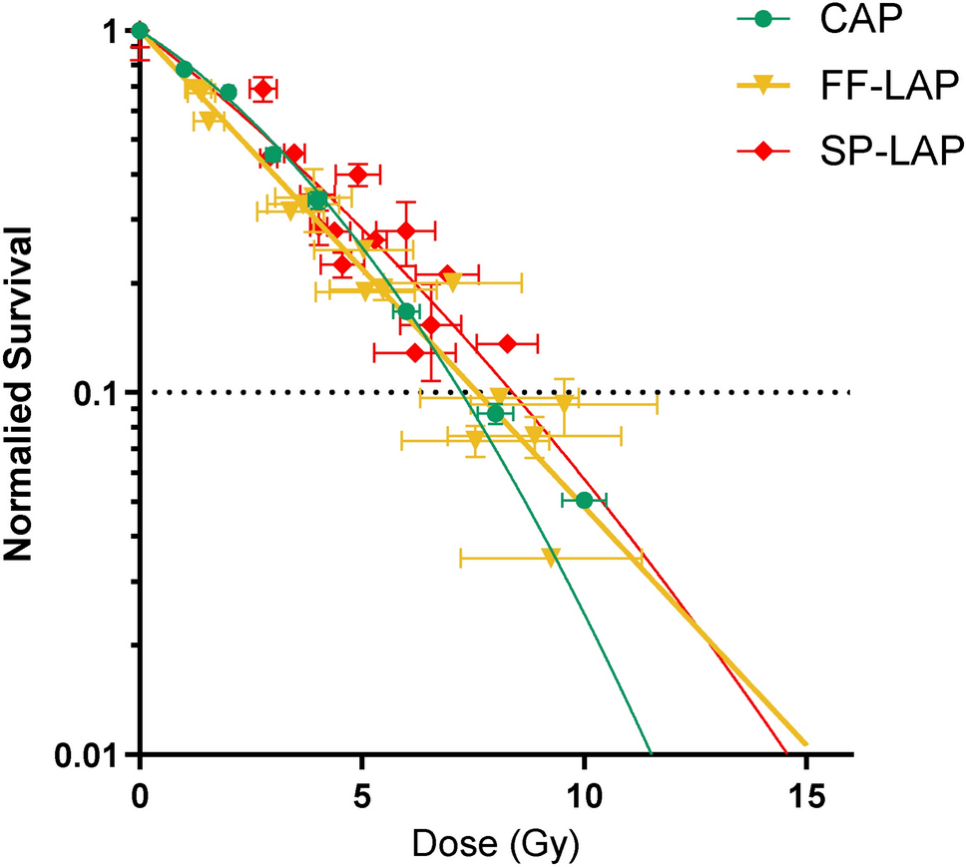
Cell survival dose-response of U87-MG cell line. Normalized cell survival resulting from exposure to increasing doses of CAP, FF-LAP and SP-LAP in U87-MG cells. Each data point represents the mean and standard deviation (SD) of three replicates obtained at least with three independent experiments. Survival curves were generated following the linear quadratic model (R squared values were 0.9756, 0.9773 and 0.9824 for CAP, FF-LAP and SP-LAP respectively).

U87-MG cells were irradiated in single pulse modality with doses ranging from 2.5 to 10.8Gy and the resulting dose-response survival curves obtained from non-clonogenic survival assays (see “Methods”). Figure 8 show a superposition between novel SP-LAP points and those already published in Bayart et al., 2019^4^. The $\hbox {D}_{10}$ values resulting from a linear-quadratic model fit do show good agreement between the three experiments, confirming the toxicity of our irradiation conditions on cancerous cells and the validity of the dose escalation scheme. The set of $D_{10}$ values across the two experiments is summarized in Table 3, along with average and instantaneous dose-rate (respectively $\dot{\overline{\textrm{D}}}$ and ${\dot{\textrm{D}}}$) and the $R^2$ value of the fit.

**Table 3:** Comparison of doses giving 10% of cell survival ($\hbox {D}_{10}$), average ($\dot{\overline{\textrm{D}}}$) and instantaneous ($\dot{\textrm{D}}$) dose-rates from CAP, FF-LAP and SP-LAP. Mean $\hbox {D}_{10} \pm$ SEM extracted from curves obtained in Figure 8 are reported. Grayed values from Bayart et al.^4^.

	Radiation quality		
	CAP	FF-LAP	SP-LAP
$\hbox {D}_{10}$ (Gy)	${7.11 \pm 0.16}$	${7.47 \pm 0.32}$	${8.20 \pm 0.43}$
$\dot{\overline{\textrm{D}}}\,\,(\hbox {Gy s}^{-1})$	5$\times 10^{-2}$	0.3 - 0.7	$10^{8}$ - $10^{9}$
${\dot{\textrm{D}}\,\,(\hbox {Gy s}^{-1})}$	5$\times 10^{-2}$	1.5$\times 10^{8}$	$10^{8}$ - $10^{9}$
$R^2$	0.9756	0.9773	0.9824

### Oxidative stress-dependent DNA damage in healthy and tumoral cell lines after exposures to SP-LAP

In the described conditions, the total dose is deposited in a time shorter than 10 ns, at a dose-rate exceeding ${10^8} \hbox { Gy s}^{-1}$. These conditions are several orders of magnitude shorter and more intense than those indicated as a threshold for FLASH effect in Bourhis et al., 2019^21^. In this single-pulse condition, dose deposition happens in a temporal span faster than the homogeneous chemical step^22^. Although the associated mechanistics is not yet known, FLASH effect has been associated to the reduction of oxidative stress in healthy tissue.

**Fig. 9:**
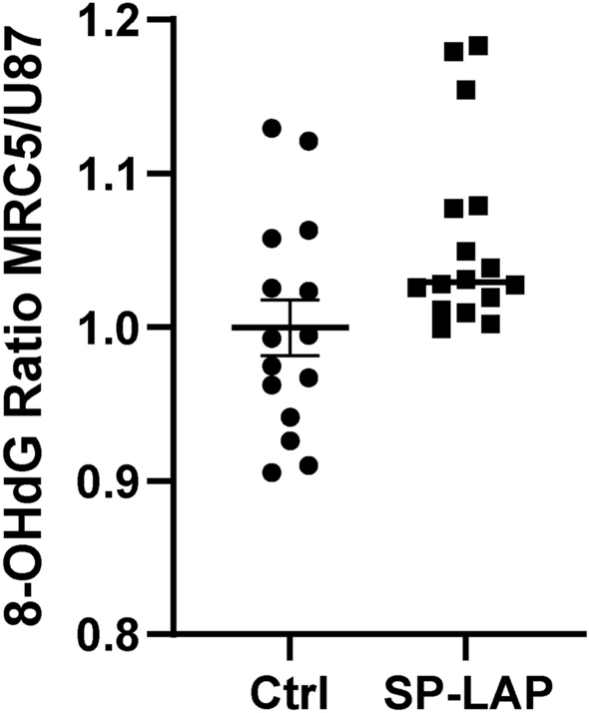
Oxidative stress-dependent DNA damage in SP-LAP irradiation. 8-Hydroxy-2’-deoxyguanosine (8-OHdG) production ratio between MRC5 and U87-MG cell lines, for both non-irradiated and SP-irradiatied cells, 1 h post irradiation. Mean doses deposited were $({3.35 \pm 0.55})$ Gy and $({2.39 \pm 0.36})$ Gy in MRC5 and U87-MG cells respectively. Mean ratio and SEM are represented, $p = 0.0076$, Kolmogorov–Smirnov test.

To address the question whether SP-LAP could have a different effectiveness to generate oxidative stress-dependent DNA damage, we aimed to measure 8-Hydroxy-2’-deoxyguanosine (8-OHdG). 8-OHdG is one of the most widely studied oxidized metabolites and is considered a biomarker for oxidative damage of DNA^23,24^. Human healthy fibroblasts MRC5 and U87-MG cells were exposed to a target dose of 2.5 Gy of SP-LAP ((3.35 ± 0.55) Gy and ${2.39 \pm 0.36}$ Gy in MRC5 and U87-MG cells respectively according to post-irradiation dosimetry). The amount of 8-OHdG was determined in cells harvested one hour later and analyzed by Elisa assay (see Methods). Using this detection method, the amount of 8-OHdG detected is inversely related to absorbance. Figure 9 shows the ratio of the values obtained with MCR5 cell line out of those of U87-MG. At the basal level, MRC5 and U87-MG cells present similar amounts of 8-OHdG, indicated by a ratio close to one (0.999 ± 0.180). However, following irradiation with SP-LAP, 8-OHdG ratio increase significantly ($p = 0.0076$, Kolmogorov–Smirnov test) to a value of 1.057 ± 0.015, corresponding to an increase of 5.7% whereas the dose applied was 14% lower compared to MRC5 cells. These result suggests that, following exposure to ultra-high dose-rate protons, the glioblastoma cell line model receives a higher fraction of DNA damage due to oxidative stress than the healthy cell model. This differential response between healthy and tumor cells observed at the level of oxidative-stress induced DNA damage may be a path to follow in order to understand healthy tissue sparing associated to Flash effect.

### Zebrafish embryo development evaluation as preclinical in vivo model for radiation research at pico2000

Radiation-induced toxicity reduction at constant dose and higher dose-rate (FLASH effect) have been exclusively observed with in vivo models (for review see Vozenin et al., 2022^25^) including mice, cats, dogs, pigs and zebrafish (*Danio rerio*). Aiming at a validation of *in vivo* irradiation of laser-driven protons, zebrafish do represent a good candidate. Zebrafish (ZF) embryos have a small size (0.5 mm to 1 mm), demanding lower proton energy and relatively small SOBP. Their embryos are fully functional organisms, with the size of organoids, for which organogenesis and morphological changes can be easily monitored during the experiment. Thus, zebrafish embryos represent a good model for radiobiological evaluation of ionizing radiation, particularly suitable for lesser penetrating laser-accelerated protons^26–29^.

**Fig. 10:**
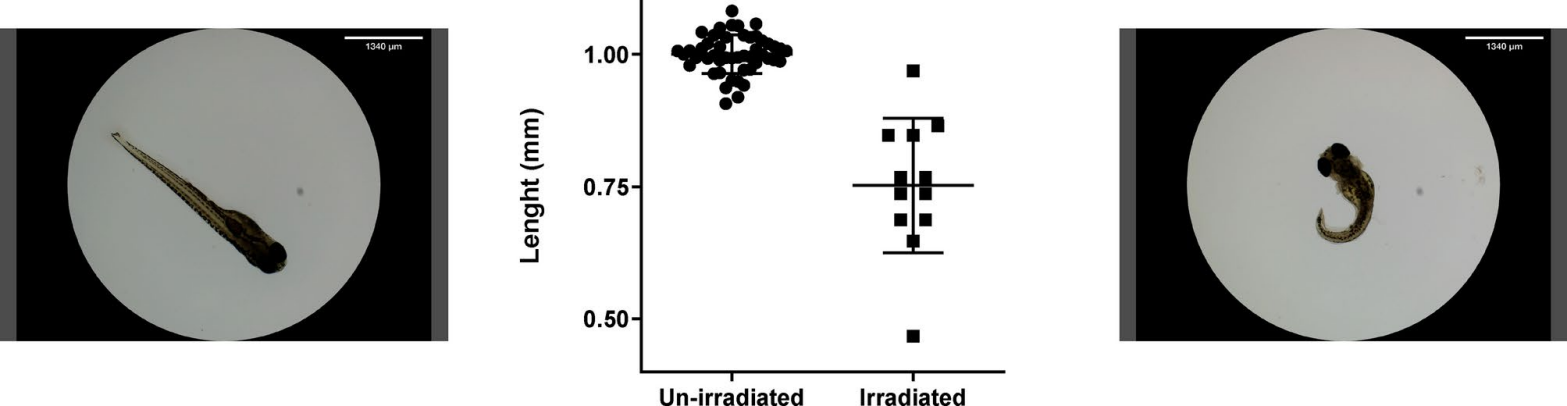
Zebrafish embryo development evaluation. Zebrafish embryos were irradiated 4 h post-fertilization, length of animals was measured 5 days post-irradiation. Example of non-irradiated (left) and of irradiated (right) fish, exhibiting spine curvature and malformations. (Center) Length of animals were expressed as ratios of the measured values for each condition out of the mean length value of non-irradiated animals. The plotted data correspond to embryos from three independent irradiations, from three independent fertilizations and days of the experimental campaign. Each data point represents one living animal. Mean dose deposited was (10.37 ± 1.42) Gy. Mean ratio and SEM are represented, $n=3$, 7 embryos per group, $p < 0.0001$ (unpaired t-test).

In order to irradiate ZF embryos, the transport line was setup to produce a uniform irradiation field over 5 mm diameter and ${600}\,{\upmu \textrm{m}}$ depth in water. A specific holder was designed to confine the embryos in close contact with the irradiation field within the irradiation field surface while preserving their survival conditions (see “Methods”). The holder allows the irradiation of several embryos at the same time. Embryos were irradiated at 4 h post-fertilization, when the embryo has a diameter of roughly ${500}\, {\upmu \textrm{m}}$. Developed fish were fixated 5 days post-irradiation, and the fish length measured as an indicator of SP-LAP toxicity (Fig. 10).

Irradiated animals exhibited changes in morphology and in the length due to spine curvature and developmental deterioration. As expected, after irradiation with SP-LAP, zebrafish embryos showed a significant decrease in total length, with a relative length corresponding to (75.20 ± 3.68)% of non-irradiated animals. As the total dose was deposited in a single bunch, our irradiation conditions fall within the current definition of FLASH. It is yet to verify if the much shorter duration and the much higher instantaneous dose rate do have measurable effects in addition to those already demonstrated. FLASH effect on zebrafish embryos was observed in similar conditions (8 Gy dose fraction at 4 h post-fertilization, developmental evaluation at 5 day post-irradiation) in Bourhis et al. 2019 paper^21^, where a 75% fish length is observed between FLASH and control condition, as opposed to 50% relative length in the conventional temporal modality. In our experiment the measured length decrease for irradiated embryos is indeed compatible with the Bourhis et al. observation of 25% of the non irradiated, for a slightly higher dose (10.37 ± 1.42) Gy).

## Discussion

Due to their favorable ballistic properties, hadrons (protons and carbon ions) represent a better alternative for the radiation therapy of solid tumors affecting organ-at-risk. The Bragg peak dose deposition profile allows the dose to be better concentrated within the target volume, reducing side effects to neighboring healthy tissues. The development of laser plasma technology and its new paradigms for protons acceleration (^30^ raise new possibilities for studying the effects of dose delivery modalities^2^. However, due to the used particle (electrons or protons) acceleration technology (laser-driven sources), the total deposited dose is delivered as a sequence of multiple ultra-short separate fractions at ultra-high instantaneous dose rates; the dose per pulse (hence the number of bunches required for a given dose) and the effective repetition rate (hence the total irradiation time) depend on technical choices or limitations at the facility^1,5,15,31–34^. In the past we explored the radiobiological impact of the repetition rate of bunches and we showed that, at constant dose, the temporal dose deposition modality of LAP is a key parameter in determining cancer cells response^4^.

Since the first proof of concept of irradiating cells with LAP^15,33,35^ we have been able to explore the biological impact of laser-accelerated protons of highly resistant glioblastoma cells, for which proton therapy is one of the main indications. Cell survival of U87-MG glioblastoma cell line showed, as expected, similar effectiveness of single-pulse-LAP (SP-LAP), compared to fast fraction-LAP and conventional beams, to induce cell killing (Fig. 8). Indeed, we showed that this cell line was unsensitive to the temporal parameter of dose deposition resulting from the absence of functional PARP1 protein which is probably responsible of its high radioresistance. We also investigated the ability of laser-accelerated protons to generate DNA double-strand breaks (DSBs); it is confirmed that LAP and conventional beams have similar effectiveness, although a number of non-significant divergences were reported (mainly arising from differences in experimental procedures and endpoints).

Flash effect and related healthy tissue sparing have been reported to be linked to reduced production of reactive oxygen species (ROS)^22,36^.

In this study, the high charge generated by the high energy pico2000 laser, using the well-established TNSA acceleration technique, enabled irradiation conditions compatible with the requirements of the FLASH protocol. Owing to the high energy/low repetition rate configuration, the FLASH condition was reached within a single 1 ps laser pulse; proton bunches duration is shorter than 10 ns, which implies a dose-rate exceeding $10^{8}\,\hbox {Gy s}^{-1}$.

The use of a quadrupole transport line, enabled to control the deposited dose within the single proton pulse. In our setup, a moving scattering filter installed after the second magnetic focusing element was used to adjust the diffusion center of proton emission, thereby affecting the particle density at the irradiation plane. This technique enables dose escalation, even with the ultra-fast nature of laser-driven acceleration mechanism. Additionally, the two focusing elements naturally influence the particle spectrum by limiting the low energy end, thus enhancing the dose contribution from the central portion. As a result, this effect produced a more uniform SOBP through the 1 mm thick biological sample.

We demonstrated how the temperature of the TNSA proton emission could be used as a parameter to fit the available diagnostics on the proton transport line. In fact the otherwise non-optimal acceleration repeatability proved to loosely depend on temperature and strongly on the amount of accelerated charge. The Analysis protocol we set up, not only enabled a precise reconstruction of the dose within the target volume, but also to extract beam parameters otherwise non accessible. This strategy will be of use for future experiments where TNSA accelerated protons will be used for practical applications, most importantly for laser-driven single-pulse FLASH irradiation condition.

Notwithstanding the challenges of retrieving irradiation conditions post-irradiation, our experiment highlights a fundamental limitation of laser-driven single-pulse FLASH: it is not possible to monitor, and therefore to control, the total deposited dose before the irradiation is completed.

The experimental evidence we gathered under laser-driven FLASH conditions was used to explore whether induction of oxidative stress-dependent DNA damage could differ between healthy and tumor cells under these irradiation modalities. By comparing the ratios between the two cell types, we observed that oxidative stress-dependent DNA damage was equivalent in non-irradiated cells; however it was higher in U87-MG glioblastoma cells than in healthy MRC5 fibroblasts (Fig. 9). This result suggests that applying SP-LAP may be able to generate a greater amount of oxidative stress-dependent DNA damage in tumor model than in a healthy one. This hypothesis aligns with recent data indicating that FLASH irradiation produces significantly more ROS than conventional irradiation, which can be eliminated much more efficiently in normal tissues during steady-state metabolism than in tumors.

## Methods

### TNSA particle source on pico2000

**Fig. 11:**
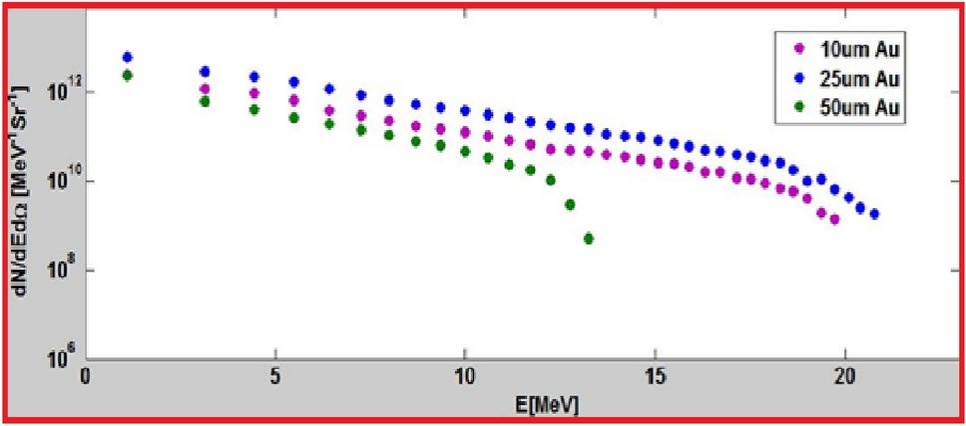
Proton spectra extracted from F. M. Perozziello thesis (Fig. 58, p. 78)^37^, performed on the pico2000 interaction chamber in similar condition as the present study.

Studies on TNSA acceleration conditions using the pico2000 laser system were performed during previous experiments, as shown in Fig. 11.

**Fig. 12:**
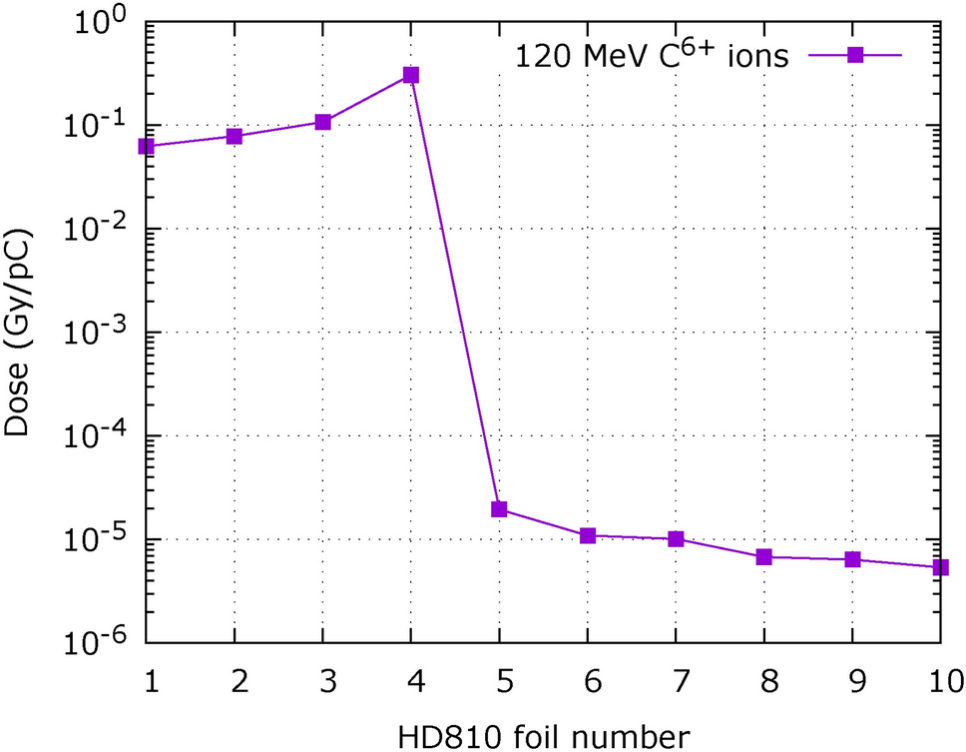
Dose deposited by ${120}\,\hbox {MeV C}^{6+}$ ions in a HD810 RCF stack.

According to Fig. 11, gold targets 10 ${\upmu }$m and 25 ${\upmu }$m showed similar performance in terms of charge and cut-off energy; for the present study a thickness of 12.5 ${\upmu }$m was chosen as the closest to best acceleration conditions among the available target thicknesses. Along with protons, heavier ion species (carbon, oxygen, ...) are usually also present among the particles driven by the plasma expansion in vacuum. In order to verify their possible impact on our experiment we simulated the propagation of a mono-energetic $\hbox {C}^{6+}$ ions beam at 120 MeV through the stack used for spectral measurement and through the transport line. Monte Carlo simulation show (Fig. 12) an important dose deposition limited to the first four radiochromic films, which are sufficient to stop the ions under analysis.

Similar simulations, performed with the complete magnetic transport line for irradiation in vitro, show that ${120}\,\hbox {MeV C}^{6+}$ ions are stopped within the protection layer of the first EBT-XD film. Considering the exceptional nature of the simulated source, monochromatic at the highest possible energy, the contribution of heavy ions was henceforth neglected.

### Dosimetry

Spatial dose distribution is measured by means of radiochromic films (Ashland corp.) type EBT-XD. Film dose response was calibrated to water equivalence on a medical protontherapy line (Centre de Protonthérapie d’Orsay, 91898 Orsay, France) with proton beam energy degraded to 20 MeV. The calibration procedure is the same followed in^5,38^.

The response of each RCF in the line is corrected for quenching effect using the curve provided by Anderson et al.^39^, weighted following the spectral distribution provided by Monte Carlo simulation in the applicable configuration and temperature. A systematic correction factor comes for RCF quenching at the used calibration energy, which accounts for a 6.5% dose excess when the indicated procedure is used. See following section for further details.

### Monte Carlo simulation

The proton beamline is modelled using the Monte Carlo toolkit Geant4, where quadrupoles are represented by measured field maps. The possibility of simulating magnetic optics with TNSA sources and energies using Monte Carlo methods was studied in greater detail and validated in Cavallone et al., 2019^40^ by comparing it with codes that account for space charge.

Monte Carlo simulations are run with a point proton source with an exponential spectrum as in equation (1), where lower and higher cutoffs matching experimental values. The lower cutoff is set to 1 MeV, which is well below the minimum energy needed to reach the first radiochromic film in absence of a scattering filter; while the maximum is set to 19.5 MeV, which corresponds to the highest measured energy. Source has isotropic angular distribution up to $\theta _{max} = {100}\,\hbox {mrad}$; source temperature $\hbox {E}_0$ and effective charge $\hbox {Q}_{0}^{*}$ are varied to match dosimetry measurement.

**Fig. 13:**
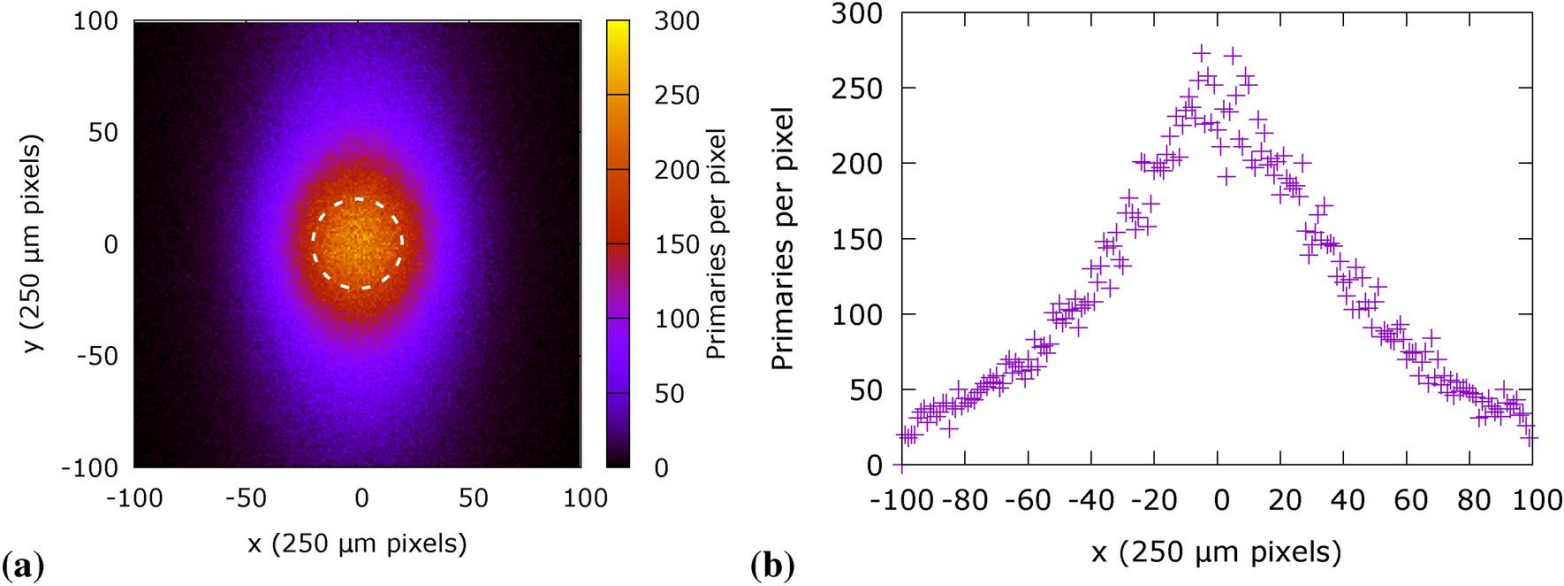
Number of primaries in a simulation, map (**a**) and center cut-out (**b**) reaching the first RCF in a simulation with ${10}\,\hbox {pC}^{*}$ of primaries. There is a minimum of ${2}\times 10^{3}\,\hbox {primaries/mm}^{2}$ (average: ${3.4}\times 10^{3}\,\hbox {primaries/mm}^{2})$, resulting in a total of 269512 primaries in the 1 cm diameter ROI.

The total proton charge on the test shot was estimated to be $\text {Q}_0 = {157}\,\hbox {nC}$, obtained from the exponential fitting algorithm described in^41^. The effective charge ratio (i.e. the total charge within the input acceptance angle of the first quadrupole) is calculated by numerical integration from fitted curves in Figure 2 and found to be $\textrm{Q}_{0}^{*} = 0.2\,\textrm{Q}_{0} \simeq {30}\hbox {nC}^{*}$ which is used as an order of magnitude of the number of particles entering the transport line. This figure does not take in account the efficiency of the particle transport, nor the actual charge within the ROI on the biological sample. Given the numerical complexity of the transport line, the number of primaries in the Monte Carlo simulation is reduced to ${10}\,\hbox {pC}^{*}$ (roughly a factor $10^{-3}$ of the experimental charge) and then the simulation results scaled to ${1}\,\hbox {nC}^{*}$. A test simulation in the dose escalation configuration shows that, on the average, 200 primaries are recorded per each 250 ${\upmu }$m pixel at the first RCF (Fig. 13) for each ${10}\,\hbox {pC}^{*}$ at the source.

### EBT-XD modelling and spectral-related corrections

EBT-XD radiochromic films are represented in the simulation as composed by two 125 ${\upmu }$m thick Mylar foils ($\rho ={1.4}\,\hbox {g cm}^{-3}$) and a 28 ${\upmu }$m thick sensitive layer ($\rho ={1.15}\,\hbox {g cm}^{-3}$, atomic composition: 51.1% Carbon, 8.8% Hydrogen, 0.6% Litium, 32.8% Oxygen, 6.7% Aluminum).

**Fig. 14:**
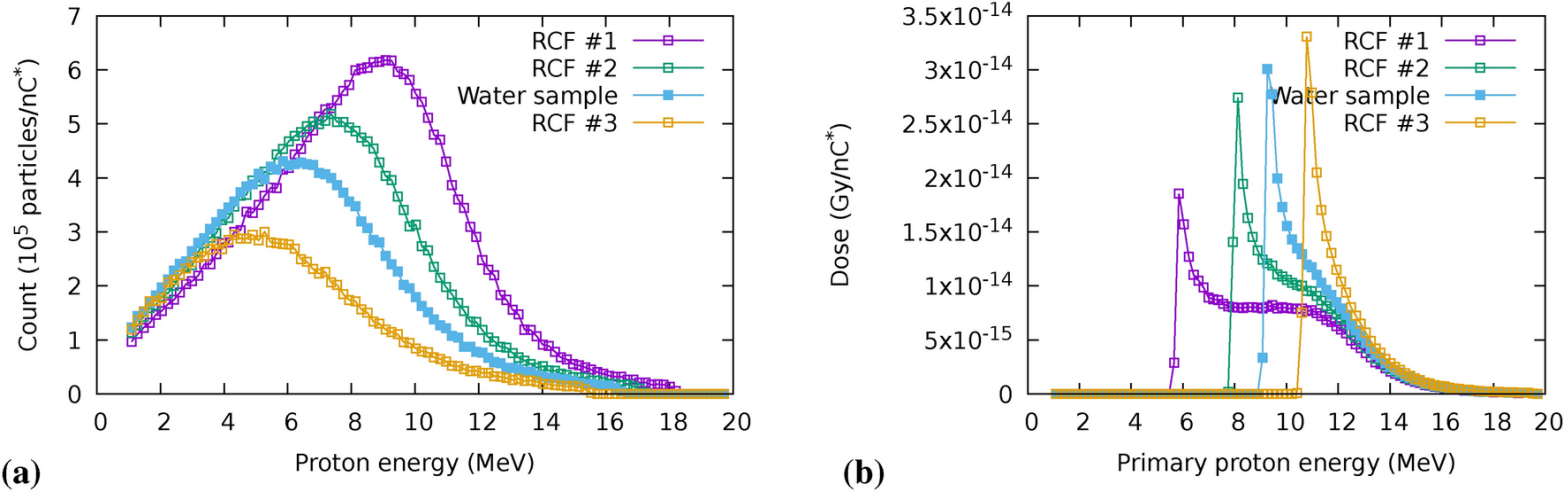
Spectral distribution of primary particles (**a**) and contribution to deposited dose of spectral components from the exponential spectrum of the proton source (**b**) at sensitive volumes in the target, within a 10 mm diameter ROI. Simulation in dose escalation configuration with $\Delta z={275}\,\hbox {mm}$ (Fig. 3a).

The evolution of the proton spectrum is recorded throughout the Monte Carlo simulation along with dose deposited in the sensitive (radiochromic films and water) volumes. An example of the spectral profile and spectral components contribution in a specific beam line configuration and source temperature (T=3 MeV, dose escalation $\Delta z={275}\,\hbox {mm}$) is presented in Fig. 14. Peak energy in the volume shifts from higher to lower energies following the propagation of the particle beam through matter. Proton with energies between 5.86 MeV to 10.7 MeV (Fig. 14b) have their Bragg peak in the biological target assembly. Spectral profiles at RCF are used for calculating the dose to water equivalence factor (DWE) and the quenching correction factor (QCF).

The DWE factor corrects for the difference between the Monte Carlo-simulated energy deposition in the RCF foil (average density $\rho ={1.375}\,\hbox {g cm}^{-3}$) and the water equivalent calibration that is performed on the medical accelerator. DWE in simulated RCF is calculated by weighting dose deposited to the RCF sensitive volume by the spectral-dependent correction factor, hence:

6 $$\begin{aligned} \displaystyle DWE = \frac{1}{\sum _i N\left( E_i \right) } \sum _{i} N \left( E_{i} \right) \frac{ \left( \frac{dE}{dz} \right) ^{(Water)}_{\left( E=E_i \right) }}{\left( \frac{dE}{dz} \right) ^{(Mylar)}_{\left( E=E_i \right) }} \end{aligned}$$

which represent the weighted sum of the ratio between the energy loss to water and the energy loss to mylar at a given energy over the simulated spectral distribution. The DWE is used for converting the simulated dose deposition to the dose recorded by RCFs during the experimental procedures.

The QCF takes in account the under estimation of dose deposited by high-LET radiation qualities in radiochromic films. Following Anderson et al.^39^, a correction factor $\eta _{LET}$ is introduced, representing the ratio between RCF reading and ionization chamber reading as a function of LET:

7 $$\begin{aligned} \eta _{LET} = -0.0251\cdot LET\left[ \text {keV} {\upmu }m^{-1} \right] + 1.02. \end{aligned}$$

The overall correction factor QCF is then calculated by averaging the deposited energy per energy band corrected by $\eta _{LET}$ over the simulated spectral distribution, hence:

8 $$\begin{aligned} QCF = \frac{1}{\sum _i D\left( E_i \right) N\left( E_i \right) } \sum _i \frac{D\left( E_i \right) N\left( E_i \right) }{\eta \left( E_i \right) } \end{aligned}$$

The QCF is used to correct the integrated dose read from the irradiated RCFs.

**Table 4:** Spectrally dependent correction factors at a source temperature of 3 MeV in the conditions of Fig. 14.

	Dose to Water Equivalence	Quenching Correction Factor
RCF 1	0.976	1.175
RCF 2	0.973	1.182
Sample	1	
RCF 3	0.971	1.189

Since both corrections (DWE, QCF) are spectrum dependent, a set of correction is calculated for each temperature and applied to experimental data before fitting. As an example, correction values for one specific configuration and temperature (dose escalation, $T={3}\,\hbox {MeV}$) are shown in Table 4.

### Radiobiology procedures

#### Cell culture

The human glioblastoma cell line U87-MG was cultured in Dulbecco’s modified Eagle’s minimum medium with Glutamax (Thermo Fisher Scientific). Cells were grown as monolayers, supplemented with 10% fetal calf serum (PAA) and 1% penicillin and streptomycin (ThermoFisher Scientific) in plastic tissue culture disposable flasks (TPP) at ${37}^{\circ }\,\hbox {C}$ in a humidified atmosphere of 5% $\mathrm {CO_2}$ in air.

#### Cell holder

**Fig. 15:**
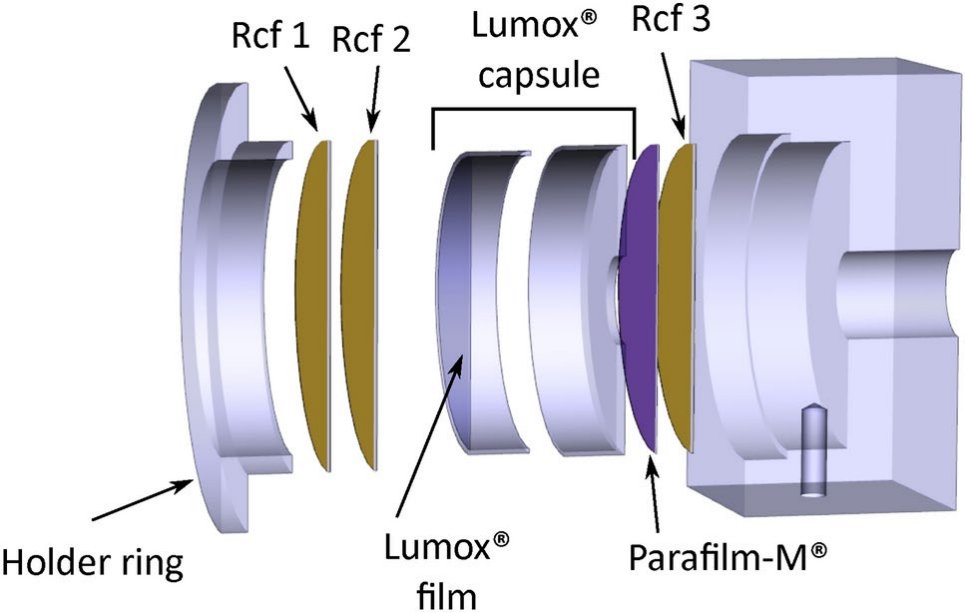
Exploded view of the holder developed for the Lumox® dish-35 cell holders.

The Lumox® dish-35 cell containers are installed on the irradiation line in a custom mount shown in Fig. 15.

An holder ring is used to keep in place the stack composed by one or two front RCFs, the Lumox® capsule and an additional back side RCF. A 10 mm hole is drilled in the bottom of the Lumox® box, and sealed with Parafilm-M® to improve the response on the back-side RCF. The front Lumox® film (where cell culture is deposited) has a measured density of ${1.10}\,\hbox {g cm}^{-3}$ and a thickness of 50 ${\upmu }$m. Parafilm-M® has a measured density of ${0.922}\,\hbox {g cm}^{-3}$ and a thickness of 127${\upmu }$m.

#### Cell survival assay

The cell containers used for irradiation were Lumox® dish-35 (SARSTEDT) exhibiting a 25 ${\upmu }$m thick Lumox® bottom face. Depending on beam transverse profile and position observed on radiochromic films a 1 cm circular area was delimited on the internal face of the Lumox® membrane, where $3 \times 10^4$ cells were seeded. Cells were let grow overnight in 200 ${\upmu }$L of medium. To generate dose-response survival curves U87-MG cell lines were subjected to doses varying from 2.5 Gy to 10.8 Gy with single bunch of laser driven protons. Survival curves resulting from irradiation with CAP or fast-fractionation LAP were retrieved from^4^. After exposure to ionizing radiations, cells were incubated for 3 h in standard conditions. Cells were harvested with Accutase (Merck), dispatched into 3 different wells of 12-well plates (TPP) in 2.5 mL of medium and grown for five generations corresponding to 6 d for U87-MG cell line. Cells were harvested with Accutase which was then inactivated using an equal volume of 1*X* PBS (ThermoFisher Scientific) supplemented with 10% fetal calf serum. The final volume was adjusted to 1 mL with 1*X* PBS and 200 ${\upmu }$L of each well were dispatched into a non-sterile U-bottom 96-well plate (TPP). In each well, 2 ${\upmu }$L of a propidium iodide solution (Sigma, 100 ${\upmu }$g/mL in 1X PBS) were added just before flow cytometry counting. Cell acquisition and data analysis were performed using Guava^®^ and GuavaSoft (Merck) and then GraphPad Prism software.

#### Oxidative stress-dependent DNA damage analysis

To perform oxidative stress-dependent DNA damage analysis, ${6\,\times \,10^{4}}$ cells from the MRC5 healthy cell line and U87-MG glioblastoma cell line were seeded in the same cell container, as describe above. Cells were irradiated at a target dose of 2.5 Gy, harvested one hour post-irradiation and immediately frozen in liquid nitrogen before being stored at ${-80}^{\circ }\hbox {C}$. Oxidative stress-dependent DNA damage (8-hydroxyguanosine, 8-OHdG) measurement was performed using DNA damage ELISA kit (Enzo) following manufacturer protocol. Absorbance at 450 nm was measured using an EnSpire Plate reader (PerkingElmer), and the data were analyzed using GraphPad Prism software.

#### Zebrafish holder

**Fig. 16:**
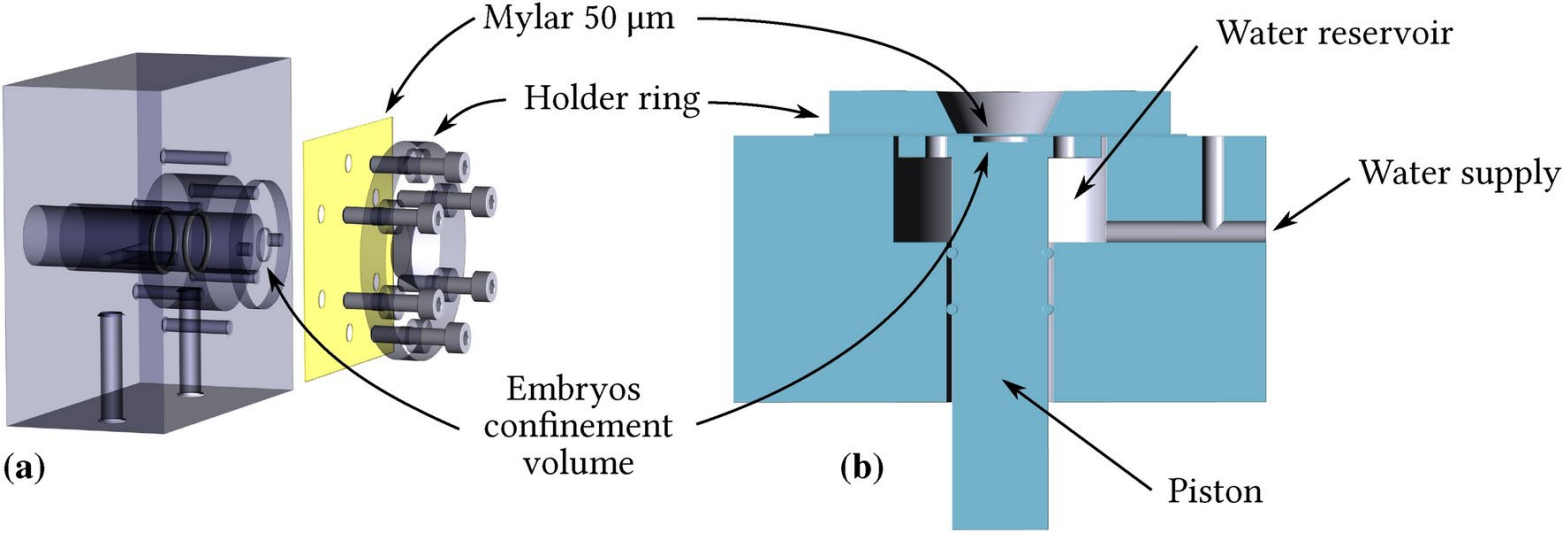
Exploded view (**a**) and sagittal cut-out (**b**) of the holder developed for the irradiation of Zebrafish embryos. The piston run through the water reservoir is visible, for switching between storage and irradiation configuration.

Owing to the limited available kinetic energy in the proton beam, a specific holder (Fig. 16) for zebrafish embryos was designed and fabricated. The embryos are kept in a water reservoir before irradiation; the holder is kept horizontal (as in Fig. 16b) and the embryos are let sink in a 600 ${\upmu }$m deep notch on the top of the piston. At the moment of irradiation the piston is pushed upwards towards the Mylar water/air separation. In this condition embryos are confined in the inner cylinder, which makes possible to put the holder in vertical position (as in Fig. 16a) for installation on the irradiation beam-line.

#### Zebrafish embryos experiments

For studies in vivo, wild-type (WT) zebrafish were bred in LOB fish facility (École Polytechnique). All in vivo experiments on zebrafish were performed on embryos below 5 days post-irradiation. Zebrafish embryos were obtained by natural spawning of WT fish. Fertilized WT zebrafish eggs were incubated at ${28}^{\circ }\hbox {C}$ until 5 days post-irradiation. Irradiation was performed 4 h post-fertilization at a target dose of 10 Gy SP-LAP and FLASH (Oriatron, Institut Curie, as control) in zebrafish holder. For FLASH irradation, zebrafish holder was placed in a water tank. Embryos were fixed 5 days post-irradiation with a solution of paraformaldehyde (4% final concentration for one hour) before microscopic analysis (Evos XL Core Cell Imaging System; Thermo Fisher Scientific). Fish length was measured using ImageJ 1.X. software.

## Data Availability

The datasets used and/or analysed during the current study available from the corresponding author on reasonable request.
